# A Convolutional Neural Network Architecture for Segmentation of Lung Diseases Using Chest X-ray Images

**DOI:** 10.3390/diagnostics13091651

**Published:** 2023-05-08

**Authors:** Adel Sulaiman, Vatsala Anand, Sheifali Gupta, Yousef Asiri, M. A. Elmagzoub, Mana Saleh Al Reshan, Asadullah Shaikh

**Affiliations:** 1 Department of Computer Science, College of Computer Science and Information Systems, Najran University, Najran 61441, Saudi Arabia; aaalsulaiman@nu.edu.sa; 2 Chitkara University Institute of Engineering and Technology, Chitkara University, Rajpura 140401, Punjab, India; sheifali.gupta@chitkara.edu.in; 3 Department of Network and Communication Engineering, College of Computer Science and Information Systems, Najran University, Najran 61441, Saudi Arabia; meabdullah@nu.edu.sa; 4 Department of Information Systems, College of Computer Science and Information Systems, Najran University, Najran 61441, Saudi Arabia; msalreshan@nu.edu.sa (M.S.A.R.); asshaikh@nu.edu.sa (A.S.)

**Keywords:** chest X-ray (CXR), k-fold validation, cancer, healthy, segmentation, convolutional neural network model, lung diseases

## Abstract

The segmentation of lungs from medical images is a critical step in the diagnosis and treatment of lung diseases. Deep learning techniques have shown great promise in automating this task, eliminating the need for manual annotation by radiologists. In this research, a convolution neural network architecture is proposed for lung segmentation using chest X-ray images. In the proposed model, concatenate block is embedded to learn a series of filters or features used to extract meaningful information from the image. Moreover, a transpose layer is employed in the concatenate block to improve the spatial resolution of feature maps generated by a prior convolutional layer. The proposed model is trained using k-fold validation as it is a powerful and flexible tool for evaluating the performance of deep learning models. The proposed model is evaluated on five different subsets of the data by taking the value of k as 5 to obtain the optimized model to obtain more accurate results. The performance of the proposed model is analyzed for different hyper-parameters such as the batch size as 32, optimizer as Adam and 40 epochs. The dataset used for the segmentation of disease is taken from the Kaggle repository. The various performance parameters such as accuracy, IoU, and dice coefficient are calculated, and the values obtained are 0.97, 0.93, and 0.96, respectively.

## 1. Introduction

Lung disease refers to medical conditions that affect the respiratory system, such as asthma, bronchitis, emphysema, and lung cancer. Treatment options depend on the type of lung disease and may include medications, surgery, and lifestyle changes. Accurate diagnosis and classification of lung disease are often best conducted by a pulmonologist after a combination of laboratory, imaging, and/or pulmonary function tests [1].

Medical imaging techniques, such as chest X-ray (CXR) and computed tomography (CT), are commonly used to visualize the internal structures of the lungs. The interpretation of these images requires the accurate delineation of lung structures from other surrounding tissues and structures [2]. Segmentation of the lungs can aid in the diagnosis of various lung diseases, including pulmonary nodules, lung cancer, emphysema, and pulmonary fibrosis. However, the manual segmentation of lungs is a time-consuming and labor-intensive process, which can lead to inter- and intra-observer variability. Computer-aided lung segmentation is a crucial step in the diagnosis and treatment of lung diseases, but the segmentation of lungs is a difficult task because of the brightness issues and surrounding structures and the presence of bones and other structures that can obscure the lungs [3].

Overall, accurate and efficient lung segmentation is essential for the diagnosis, treatment, and monitoring of lung diseases. In recent years, deep learning techniques, particularly convolutional neural networks (CNNs), have shown remarkable success in medical image analysis tasks, including segmentation. Deep learning models can learn to automatically extract relevant features from medical images and produce accurate segmentation results. Moreover, deep learning-based segmentation methods can reduce the time and effort required for manual segmentation [4]. In this study, a deep learning-based approach for the segmentation of lungs from CXR images is proposed. The major contributions of the study are as follows:

1. A deep learning model having a series of convolution blocks and concatenate blocks has been proposed for lung segmentation using chest X-ray images. The dataset used for the segmentation of disease is taken from the Kaggle repository.
2. In the proposed model, concatenate block is embedded to learn a series of features used to extract meaningful information from the image. Moreover, a transpose layer is employed in the concatenated block to improve the spatial resolution of feature maps generated by a prior convolutional layer.

The proposed model is trained using k-fold validation by taking the value of k as 5 to obtain the best-optimized model. The performance of the proposed model is measured in terms of IoU, Dice coefficient, and accuracy for hyper-parameters, namely batch size as 32, optimizer as Adam and 40 epochs.

The rest of the study is laid out as follows: Section 2 presents the literature review, Section 3 displays the input dataset, Section 4 presents the suggested CNN model for lung segmentation, Section 5 displays the results, and Section 6 offers a summary and conclusion.

## 2. Literature Review

Here, a review of some of the most groundbreaking studies on model checking and lung segmentation of chest X-ray (CXR)/computed tomography (CT) images is performed. A brief discussion of the difficulties, biases, and possible flaws is also studied. Souza et al. [2] presented the segmentation operation using the AlexNet-based model for the initial segmentation and the ResNet18 model for the reconstruction and performance of the final segmentation task. They worked on the MC database with 138 CXR images and obtained a value of accuracy of 96.9% and a dice score of 93.56%. Kim et al. [3] performed the segmentation of chest X-ray images using U-net architecture. They worked using three databases, namely Montgomery (MC), Japanese Society of Radiological Technology (JSRT), and Shenzhen, with 138, 154 and 662 images, respectively. They obtained a value of dice score of 94%. Selvan et al. [5] presented the U-Net model for segmentation using variational data imputation. The authors performed data augmentation on the publicly available CXR datasets obtained from Shenzhen and Montgomery hospitals using 704 CXR images and obtained a value of accuracy of 88%. Lascu et al. [6] presented the transfer-learning-based ResNet101 model to classify pneumonia and healthy lungs using CXR images. Rashid et al. [7] presented a CNN network using morphological operations for the segmentation of lungs from CXR images. They worked on three different datasets, namely JSRT, MC, and local database consisting of 247, 138, and 37 CXR images, respectively. The authors obtained values of dice score and accuracy of 95.1% and 97.1%, respectively. Gaal et al. [8] used Attention U-Net architecture with Contrast Limited Adaptive Histogram Equalization (CLAHE) for the pre-processing of data. They used 247 CXR images and obtained a dice score value of 97.5% using the JSRT dataset. Reza et al. [9] proposed the TransResUNetencoder–decoder model for lung segmentation. They used the flood fill algorithm for hole filling. The operation was performed on the MC database with 138 images and obtained a value a dice score of 97.6%. Teixeira et al. discussed the impact of inventing a Chest X-ray images database from multiple resources. The achieved results measured a Jaccard distance of 0.034 and a Dice coefficient of 0.982 [10]. Gordienko et al. [11] presented a U-Net-based CNN model for the segmentation of lungs from CXR images. They used the JSRT database with 247 CXR images. They eliminated bone shadow from the CXR images using semantic segmentation. Islam et al. [12] presented a U-Net algorithm for the segmentation of lungs from CXR images. They performed work using two datasets, namely Montgomery and Shenzhen, consisting of 138 and 615 X-ray images. They obtained the value of a dice score of 98.6%. They achieved a value of accuracy of 94.9%. Song et al. [13] presented CNN for the classification of lung cancer. They worked on 4581 CT images and obtained a value of accuracy of 84.15%.

Overall, the literature suggests that accurate and reliable lung segmentation remains a crucial area of research with significant clinical implications and that ongoing advancements in deep learning and computer vision are likely to continue to drive progress in this field [14,15]. Therefore, in the proposed research, concatenate block is embedded to learn a series of filters or features used to extract meaningful information from the image. Moreover, the trans-pose layer is employed in the concatenated block to improve the spatial resolution of feature maps generated by a prior convolutional layer.

## 3. Input Dataset

**Figure 1:**
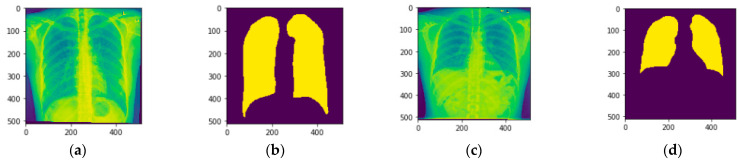
Chest X-ray Samples (**a**) Image 1, (**b**) Image 1 mask, (**c**) Image 2, and (**d**) Image 2 mask.

Kaggle [16,17] is the source for the chest X-ray dataset. Files in various folders hold X-rays and masks of the chest. The test dataset has 96 CXR images with their respective masks and the training dataset contains 704 CXR images with 704 masks and clinical readings in a text file. Lung tissue samples taken from a chest X-ray are illustrated in Figure 1. Figure 1a,c show the original photos, while Figure 1b,d show the original images’ masks.

## 4. Proposed CNN Model for the Segmentation of Lungs

**Figure 2:**
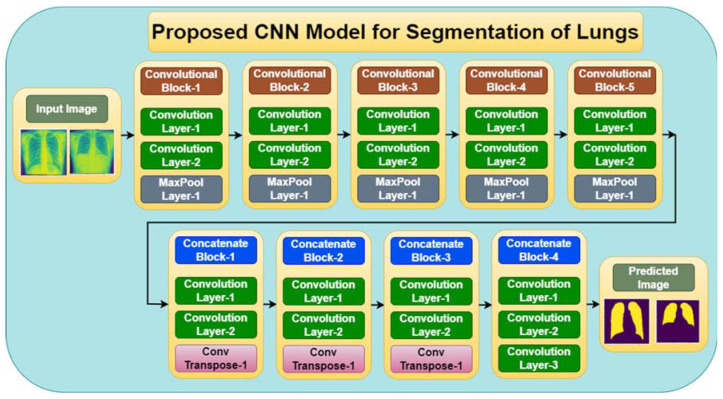
Proposed CNN model for Lung Segmentation.

The proposed CNN architecture is composed of multiple layers, with each layer playing a specific role in the overall model. Figure 2 shows the proposed CNN model for lung segmentation that is made up of convolutional blocks and concatenate blocks. A series of convolutional layers in the concatenated block learn a series of filters or features used to extract meaningful information from the image. In summary, using more convolutional layers in a CNN can help improve the performance of image classification or segmentation tasks, but it is important to carefully balance model complexity with the amount of available training data and computational resources.

Table 1 describes each block, the layers used in each block, the input image size and the number of filters used at every layer, the activation function, and the number of parameters used for each convolution layer.

## 5. Results

In this section, the proposed deep learning model has been simulated with different hyperparameters such as batch size, epochs, and optimizer. The batch size is set to 32, and the simulation runs for a maximum of 40 epochs using the Adam optimizer [18,19]. The proposed model is hypertuned using the k-fold validation method with values of k as 5. Several analyses, including loss, accuracy, dice coefficient, and IoU, are carried out for training and validating chest X-ray pictures using K-fold validation.

### 5.1. K fold Validation Results

Here, five-fold cross-validation is used to assess a model’s efficacy by splitting the data into five equal halves (called “folds”), training it on four-folds, and then testing it on the remaining fold. The dataset will be divided into five equal folds, where each fold serves as the validation set in each iteration. As a model performance metric, we use the mean value of this metric over five iterations. It also helps to reduce the variance in performance estimates by averaging the performance measure over multiple test sets. Here are some possible reasons why k = 5 worked well for a particular data set:

- Suitable sample size: This is a reasonable sample size to obtain a reliable estimate of the model’s performance. This can reduce the variance of metrics.
- Generalizability: Using a value of k = 5 computes the metric using data representative of the entire dataset. This helps ensure that the evaluation metrics are generalizable and not focused on a particular subset of the data.

#### 5.1.1. Loss Results

**Figure 3:**
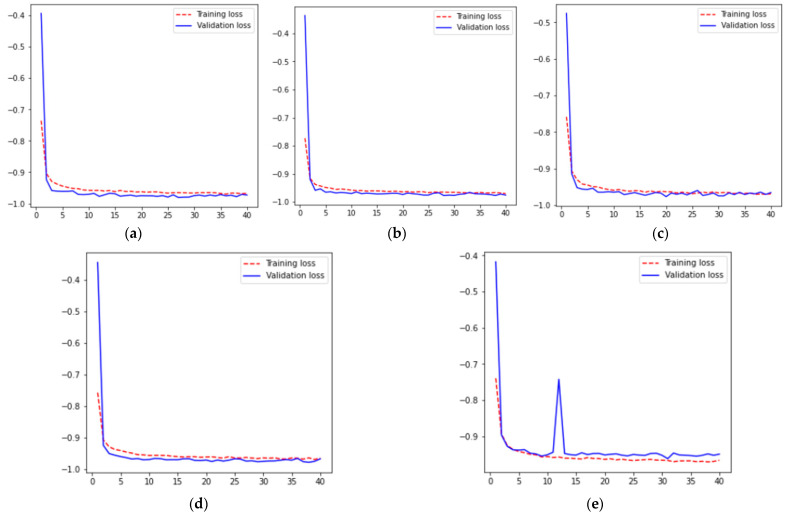
Loss Results (**a**) Fold 1, (**b**) Fold 2, (**c**) Fold 3 (**d**) Fold 4, and (**e**) Fold 5.

For five different iterations across 40 epochs, the resulting loss curves are shown in Figure 3, where Figure 3a–e represent the training and validation loss curves for values of 1, 2, 3, 4, and 5th fold validation set for k-fold cross-validation results, respectively. From these curves, it can be analyzed that for all folds, training and validation loss is decreasing from 0 to the 3rd epoch. After that loss is approximately constant from the 3rd to 40th epoch having a value of −0.95.

#### 5.1.2. IoU Results

**Figure 4:**
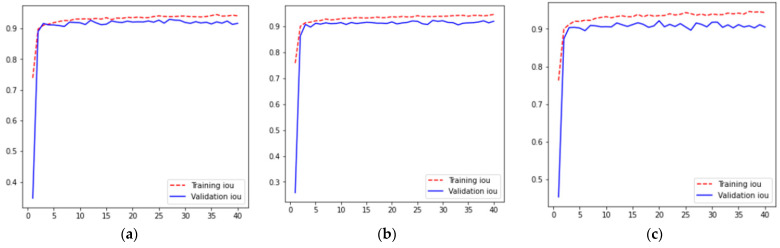

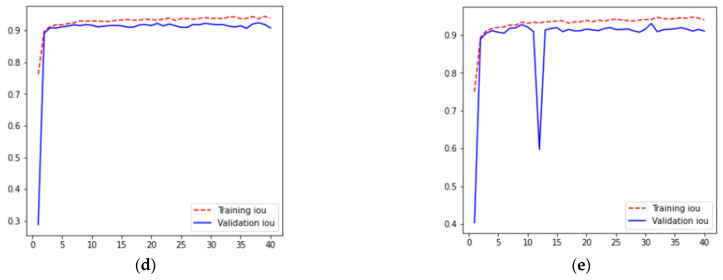
IoU Results (**a**) Fold 1, (**b**) Fold 2, (**c**) Fold 3 (**d**) Fold 4, and (**e**) Fold 5.

For five different iterations across 40 epochs, the resulting IoU curves are shown in Figure 4, where Figure 4a–e represent the training and validation IoU curves for values of 1, 2, 3, 4, and 5th fold validation set for k-fold cross-validation results, respectively. From these curves, it can be analyzed that for all folds, training and validation IoU increases from 0 to the 3rd epoch. After that, the IoU is approximately constant from the 3rd to the 40th epoch having a value of 0.93. Moreover, it can also be analyzed from Figure 4 that for all folds training IoU becomes greater than validation IoU at the 3rd epoch.

#### 5.1.3. Dice Coefficient Results

**Figure 5:**
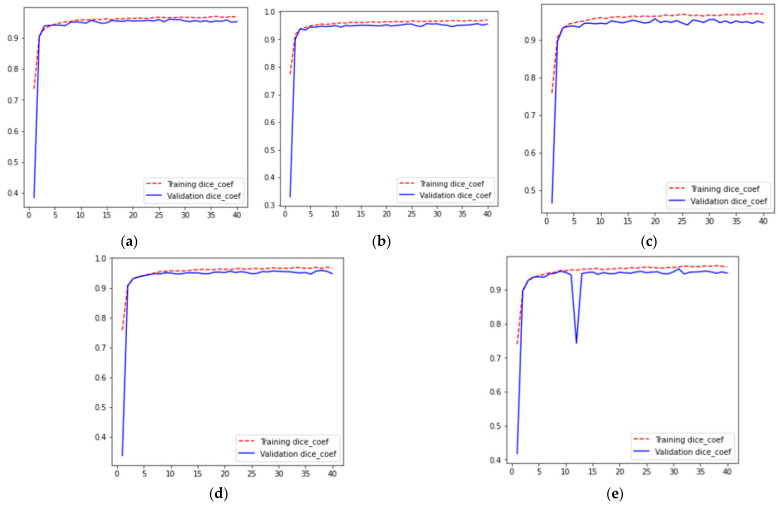
Dice Coefficient Results (**a**) Fold 1, (**b**) Fold 2, (**c**) Fold 3 (**d**) Fold 4, and (**e**) Fold 5.

For five different iterations across 40 epochs, the resulting dice coefficient curves are shown in Figure 5, where Figure 5a–e represent the training and validation dice coefficient curves for values of 1, 2, 3, 4, and 5th fold validation set for k-fold cross-validation results, respectively. From these curves, it can be analyzed that for all folds, training and validation dice coefficient increases from 0 to the 3rd epoch. After that, the dice coefficient is approximately constant from the 3rd to the 40th epoch, having a value of 0.96. Moreover, it can also be analyzed from Figure 5 that for all folds, the training dice coefficient becomes greater than the validation dice coefficient at the 3rd epoch.

#### 5.1.4. Accuracy Results

**Figure 6:**
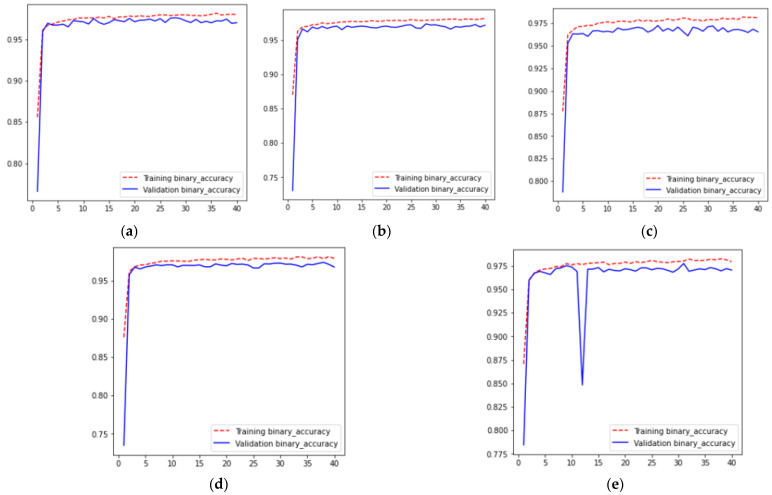
Accuracy Results (**a**) Fold 1, (**b**) Fold 2, (**c**) Fold 3 (**d**) Fold 4, and (**e**) Fold 5.

For five different iterations across 40 epochs, the resulting accuracy curves are shown in Figure 6, where Figure 6a–e represent the training and validation. Accuracy curves for values of 1, 2, 3, 4, and 5th fold validation set for k-fold cross-validation results, respectively. From these curves, it can be analyzed that for all folds, training and validation accuracy increases from 0 to the 3rd epoch. After that accuracy is approximately constant from the 3rd to the 40th epoch, having a value of 0.97. Moreover, it can also be analyzed from Figure 6 that for all folds the training accuracy becomes greater than the validation accuracy at the 3rd epoch.

At each iteration, the model is trained on four-folds and evaluated on the validation set. The performance metric is then recorded, and the process is repeated for each fold. Finally, the five performance metrics are averaged to obtain a single estimate of the model’s performance.

### 5.2. Visualization of Segmentation Results

**Figure 7:**
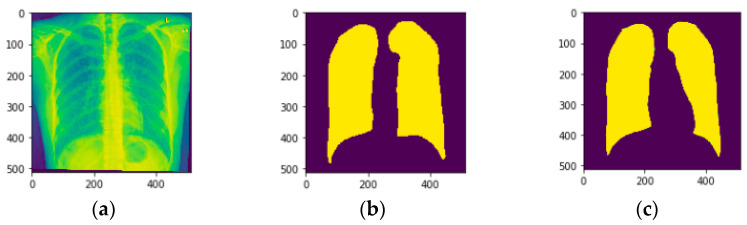
Testing Samples (**a**) Original Image, (**b**) Image mask, and (**c**) Predicted Image.

Figure 7 illustrates the testing samples of chest X-ray images in which the segmentation of the lung is performed. Figure 7a illustrates the original image sample, and Figure 7b illustrates the provided ground truth mask. Figure 7c illustrates the predicted image. It can be seen from Figure 7c that the predicted image illustrates the exact boundaries like the original image. The proposed CNN model has performed best in segmenting the chest from chest X-ray images. It is important to note that there is no single value of k that always gives the best performance for a particular dataset and model. The optimal value for k depends on factors such as the size and complexity of the dataset, the model being evaluated, and the evaluation metric used.

**Figure 8:**
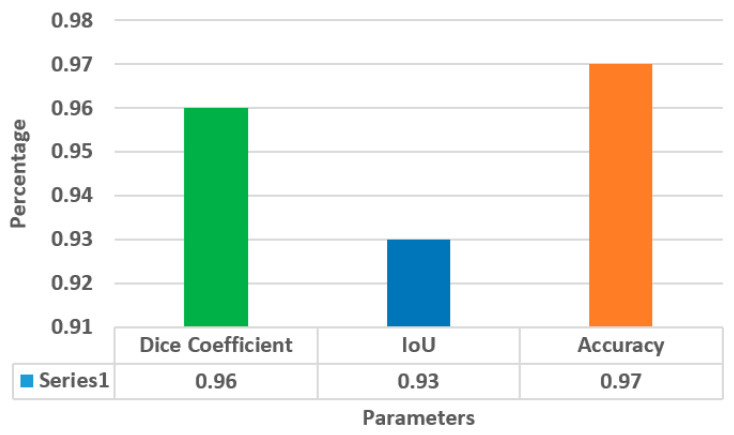
Average Values of Parameters at k = 5.

Figure 8 illustrates the average values of the dice coefficient, IoU, and accuracy on the testing dataset for k = 5. The value of accuracy is 0.97, and the proposed CNN model has outperformed in comparison with the state-of-the-art techniques, as shown in the next section. The proposed model incorporates a concatenated block to learn a set of filters or features that may be applied to an image in order to extract useful information. The spatial resolution of feature maps created by a preceding convolutional layer is also enhanced by using a transpose layer in the concatenated block.

### 5.3. Comparison with State-of-the-art

For the comparison of the proposed model with other existing techniques, the same dataset with 704 lung disease images is implemented using different transfer learning models and CNN architecture. It includes the implementation of different techniques, such as ResUNet, DenseNet121, VGGUNet and CNN, on the same dataset. Various performance parameter values for each technique and proposed model are shown in Table 2, from which it can be analyzed that the proposed model with embedded concatenate and transpose blocks has outperformed other transfer learning models and CNN architecture in terms of accuracy, dice score, and IoU.

For the comparison of the proposed model with other authors, the proposed model results have been compared with other state-of-the-art techniques shown in Table 3. It summarizes the performance parameters, including accuracy, sensitivity, specificity, dice score, and Jaccard index of different authors used for different lung disease CXR image datasets. The techniques include various deep learning architectures, such as AlexNet, ResNet, U-Net, TransResUNet, VGG, Transfer Learning, and CNN with morphological operations. The authors have used these techniques on different datasets having different numbers of CXR images or CT images. It can also be analyzed from Table 3 that the proposed model has outperformed the other state-of-the-art models in terms of all performance parameters.

Other authors are either using transfer learning models or using simple CNN architecture to segment lung disease from CXR images. No author has used CNN architecture with concatenation block and transpose layer to learn as a set of filters required to derive meaning from an image. To further enhance the spatial resolution of feature maps produced by a previous convolutional layer, a transpose layer is used in the concatenated block.

The proposed model has achieved an accuracy of 97% on a dataset of 704 images. This performance is better than most of the other techniques listed in the table.

Overall, the table shows that deep learning techniques can achieve high accuracy and performance in image analysis tasks. However, the choice of the specific architecture and the number of images used for training can significantly affect performance. Therefore, it is essential to carefully choose the appropriate architecture and optimize the hyperparameters for the specific task and dataset.

## 6. Conclusions

Medical image analysis includes the crucial duty of segmenting lung disease from chest radiographs, which aids in the diagnosis and early detection of lung disease. Lately, convolutional neural networks (CNN) and other deep learning approaches have been successfully employed to identify lung disease areas in chest radiographs. Several studies have demonstrated the effectiveness of CNN-based techniques for segmenting lung disease from chest X-rays. However, the following issues remain, such as image quality variability and similarity between normal and abnormal lung regions. Therefore, a deep learning model, having a series of convolution blocks and concatenate blocks, has been proposed for lung segmentation using chest X-ray images. In this proposed model concatenate block is embedded to learn a series of features used to extract meaningful information from the image. Moreover, a transpose layer is employed in the concatenated block to improve the spatial resolution of feature maps generated by a prior convolutional layer. An accuracy of 97% was achieved using the proposed CNN model, with very low loss. In the future, this proposed technique can help doctors diagnose diseases in the lungs and act as a second opinion tool. Further research is needed to develop more robust and effective segmentation methods for improving clinical diagnosis and treatment of lung disease. In the future, further types of layers, such as normalization layers and dropout layers, can be added to CNNs in addition to the basic layers to further enhance performance.

## Figures and Tables

**Table 1:** Parameters of the Proposed CNN model.

S. No.	Blocks	Layers	Input Image Size	Filters	Activation Function	Parameters
1		Input	512 × 512 × 3	-	-	0
2	Convolution Block-1	Conv-1	512 × 512 × 32	32	ReLU	320
		Conv-2	512 × 512 × 32	32	ReLU	9248
		MaxPool-1	256 × 256 × 32	32	-	0
3	Convolution Block-2	Conv-1	256 × 256 × 64	64	ReLU	18,496
		Conv-2	256 × 256 × 64	64	ReLU	36,928
		MaxPool-1	128 × 128 × 64	64	-	0
4	Convolution Block-3	Conv-1	128 × 128 × 128	128	ReLU	73,856
		Conv-2	128 × 128 × 128	128	ReLU	147,584
		MaxPool-1	64 × 64 × 128	128	-	0
5	Convolution Block-4	Conv-1	64 × 64 × 256	256	ReLU	295,168
		Conv-2	64 × 64 × 256	256	ReLU	590,080
		MaxPool-1	32 × 32 × 256	256	-	0
6	Convolution Block-5	Conv-1	32 × 32 × 512	512	ReLU	295,168
		Conv-2	32 × 32 × 512	512	ReLU	590,080
		MaxPool-1	16 × 16 × 512	512	-	0
7	Concatenate Block-1	Conv-1	64 × 64 × 256	256	ReLU	1,179,904
		Conv-2	64 × 64 × 256	256	ReLU	590,080
		Transpose-1	128 × 128 × 128	128	-	131,200
8	Concatenate Block-2	Conv-1	128 × 128 × 128	128	ReLU	295,040
		Conv-2	128 × 128 × 128	128	ReLU	147,584
		Transpose-1	256 × 256 × 64	128	-	32,832
9	Concatenate Block-3	Conv-1	256 × 256 × 64	64	ReLU	73,792
		Conv-2	256 × 256 × 64	64	ReLU	36,928
		Transpose-1	512 × 512 × 32	64	-	8224
10	Concatenate Block-4	Conv-1	512 × 512 × 32	32	ReLU	18,464
		Conv-2	512 × 512 × 32	32	ReLU	9248
		Conv-3	512 × 512 × 32	32	ReLU	33

**Table 2:** Comparison with existing techniques on our dataset.

Technique	Accuracy	Dice Score	IoU
ResUNet	0.96	0.95	0.92
DenseNet121	0.97	0.94	0.91
VGGUNet	0.98	0.91	0.93
CNN	0.53	0.92	0.91
Proposed CNN embedded with concatenate block and transpose layer	0.97	0.96	0.93

**Table 3:** Comparison with existing State-of-the-art techniques.

Ref/Year	Technique	Number of Images	Type of Images	Performance Parameters
[2]/2019	AlexNet ResNet18	138	CXR	Sensitivity = 97.54%, Specificity = 96.79%, Accuracy = 96.97%, Dice Score = 93.56%, Jaccard = 88.07%
[3]/2021	U-Net	662	CXR	Dice Score = 94%, Sensitivity = 94%
[5]/2020	U-Net	704	CXR	Dice = 85%, Accuracy = 88%
[6]/2021	Transfer Learning	321	CXR + CT	Accuracy = 94.9%
[7]/2018	CNN network with Morphological operations	247	CXR	Dice Score = 95.1%, Sensitivity = 95.1%, Specificity = 98.0%, Accuracy = 97.1%
[8]/2020	U-Net Architecture	247	CXR	Dice Score = 97.5%
[9]/2020	TransResUNet	138	CXR	Dice Score = 97.6%, Accuracy = 98.5%
[12]/2018	U-Net Architecture	615	CXR	Dice score = 98.6%
[13]/2017	CNN	4581	CT	Accuracy = 84.15%
Proposed Model	CNN embedded with concatenate block and transpose layer	704	CXR	Accuracy = 97% Dice Score = 96% IoU = 93%

## Data Availability

Not applicable.
